# Ophiobolin-O Reverses Adriamycin Resistance via Cell Cycle Arrest and Apoptosis Sensitization in Adriamycin-Resistant Human Breast Carcinoma (MCF-7/ADR) Cells

**DOI:** 10.3390/md11114570

**Published:** 2013-11-14

**Authors:** Wenxia Sun, Cuiting Lv, Tonghan Zhu, Xue Yang, Shanjian Wei, Jieyin Sun, Kui Hong, Weiming Zhu, Caiguo Huang

**Affiliations:** 1 Department of Biochemistry and Molecular Biology, College of Basic Medical, Second Military Medical University, 800 Xiangyin Road, Shanghai 200433, China; E-Mails: 515812385@163.com (W.S.); lvcuiting961021@126.com (C.L.); sjwei8012@hotmail.com (S.W.); jysun803@sohu.com (J.S.); 2 The Key Laboratory of Marine Drugs, Ministry of Education of China, School of Medicine and Pharmacy, Ocean University of China, Qingdao 266003, China; E-Mail: sdueduzth@126.com; 3 Key Laboratory of Combinatorial Biosynthesis and Drug Discovery, Ministry of Education, School of Pharmaceutical Sciences, Wuhan University, Wuhan 430071, China; E-Mail: yangxue@126.com

**Keywords:** Ophiobolin-O, adriamycin (ADM), drug resistance reversal

## Abstract

Multidrug-resistance is a major obstacle facing cancer chemotherapy. This paper demonstrates that novel compound Ophiobolin-O reverses MCF-7/ADR resistance to adriamycin (ADM). The IC_50_ of ADM treated MCF-7 cells was 2.02 ± 0.05 µM and 74.00 ± 0.18 µM treated MCF-7/ADR cells, about 37-fold, compared to the former. However, 0.1 µM Ophiobolin-O (less than 20% inhibition concentration) combined with ADM caused the decreased IC_50_ of ADM to 6.67 ± 0.98 µM, indicating it reversed ADM resistance of MCF-7/ADR cells (11-fold). Furthermore, Ophiobolin-O increased ADM-induced mitochondrial pathway apoptosis and G2/M phase arrest, which is partly due to the elevation level of ROS in MCF-7/ADR cells. As we described in this paper, the reversal effect of Ophiobolin-O may be due to the reduction of resistance-related protein P-Glycoprotein (P-gp, also known as MDR1) through inhibiting the activity of the multidrug resistance 1 (MDR1) gene promoter, which makes MCF-7/ADR cells more sensitive to ADM treatment. Assays in nude mice also showed that the combination of ADM and Ophiobolin-O significantly improved the effect of ADM.

## 1. Introduction

Adriamycin (ADM) is a commonly used chemotherapy drug for breast cancer. It inhibits tumor growth by inducing apoptosis, but multidrug resistance is the main reason that chemotherapy in breast cancer cells remains largely unsuccessful [1]. Identifying novel compounds that reverse drug resistance in breast cancer is urgently needed [2].

Drug resistance of tumor cells is related to the overexpression of multidrug resistance proteins. P-Glycoprotein (P-gp) is one of the multidrug resistance-related proteins and is encoded by the multidrug resistance 1 (MDR1) gene. P-gp is also the main type of drug resistance protein in MCF-7/ADR cells [3,4]. The expression of resistance proteins reduces the concentration of ADM in tumor cells, which decreases the effect of chemotherapy.

Ophiobolin-O is a novel sesquiterpene compound isolated from *Aspergillus ustus* 094102. Previous studies suggested that this compound inhibited the growth of MCF-7 cells by inducing cell cycle arrest and apoptosis [5]. Our study demonstrated that 0.1 µM Ophiobolin-O (less than 20% inhibition concentration) reverses MCF-7/ADR resistance to ADM via decreasing the expression of resistance genes, which triggered G2/M phase arrest and increased the ADM-induced apoptosis in MCF-7/ADR cells. *In vivo*, the combination of Ophiobolin-O and ADM significantly inhibited tumor growth, suggesting Ophiobolin-O as a potential agent to reverse chemotherapy drug resistance.

## 2. Results and Discussion

### 2.1. Ophiobolin-O Reversed ADM Resistance

#### 2.1.1. Ophiobolin-O Appeared to Reverse ADM Resistance in MCF-7/ADR Cells

**Figure 1:**
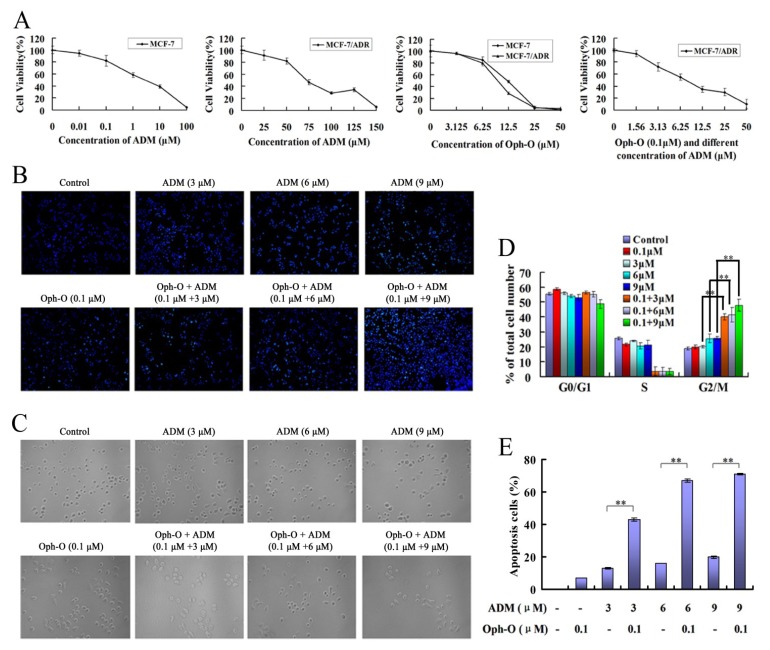
(**A**) Cell proliferation inhibition was analyzed by MTT assay, values are means ± S.D. from three independent experiments; (**B**,**C**) Cells were treated with Ophiobolin-O (0.1 µM), ADM (3 µM, 6 µM or 9 µM) alone or combination for 48 h, then nuclear morphology (**B**) or cell morphology changes (**C**) were observed. Hoechst33258 was used to stain nuclei. Representative photomicrographs of multi-groups were showed; (**D**) Harvested cells were stained using PI solution, cell cycle phase analysis was performed by using a FACScalibur flow cytometer, the percentage of multi-groups cells in different phases of the cell cycle was represented by a bar diagram; (**E**) Annexin V-FITC/PI staining for apoptosis in MCF-7/ADR cells was assessed by flow cytometry analysis, values are means ± SD from three independent experiments. * *P* < 0.05, ** *P* < 0.01 *vs.* control group.

MTT results showed that the IC_50_ of ADM at 48 h was 2.02 ± 0.05 µM and 74.00 ± 0.18 µM when ADM treated MCF-7 cells and MCF-7/ADR cells, respectively. MCF-7/ADR cells displayed approximately 37-fold elevated resistance to ADR than MCF-7 cells. On the other hand, the IC_50_ of Ophiobolin-O when treated MCF-7 was 17.86 ± 1.26 µM and 28.03 ± 0.78 µM treated MCF-7/ADR cells. However, after MCF-7/ADR cells were incubated by the combination of different concentrations of ADM and 0.1 µM Ophiobolin-O (less than 20% inhibition concentration) for 48 h, the IC_50_ of ADM decreased to 6.67 ± 0.98 µM and the reversal ratio reached 11-fold. MTT data revealed that Ophiobolin-O reversed the ADM resistance of MCF-7/ADR cells (Figure 1A).

#### 2.1.2. Nuclear Morphology Changes

Nuclear morphology of MCF-7/ADR cells was assessed by Hoechst33258 staining [6,7,8,9]. Treatment with Ophiobolin-O (0.1 µM) or ADM (3 µM, 6 µM or 9 µM) alone showed that the effect on the nuclear morphology of MCF-7/ADR cells was less than the combination of Ophiobolin-O (0.1 µM) and ADM (3 µM, 6 µM or 9 µM). The combination led to a significant chromatin condensation and fragmentation of nuclei in MCF-7/ADR cells at 48 h (Figure 1B).

#### 2.1.3. Changes of Cell Count

Cells were cultured in 6-well plates. The number of viable cells was observed with a microscope. Compared with the control group, treatment with Ophiobolin-O (0.1 µM) or ADM (3 µM, 6 µM or 9 µM) alone did not significantly decrease the cell number. However, combined treatment with Ophiobolin-O (0.1 µM) and ADM (3 µM, 6 µM or 9 µM) caused a significant reduction of the MCF-7/ADR cells’ number at 48 h (Figure 1C).

#### 2.1.4. Combination with Ophiobolin-O Caused G2/M Phase Arrest in MCF-7/ADR Cells

Flow cytometric analysis of the cell cycle showed that, compared with the control group, more cells at G2/M phase were observed when treated with 6 µM or 9 µM ADM, but there was no obvious cell cycle arrest. After treatment with Ophiobolin-O (0.1 µM) and ADM (3 µM, 6 µM or 9 µM), MCF-7/ADR cells were arrested at G2/M cell cycle phase. These results indicated that cell cycle was arrested in G2/M phase by the low concentration of Ophiobolin-O combined with ADM (Figure 1D).

#### 2.1.5. Ophiobolin-O Increased the Apoptosis Rate Induced by ADM in MCF-7/ADR Cells

Flow cytometry results showed that, compared with the control group at 48 h, Ophiobolin-O (0.1 µM) treatment caused 7.40% ± 0.77% of apoptosis; different concentration of ADM (3 µM, 6 µM or 9 µM) induced 13.09% ± 1.02%, 16.25% ± 0.98%, and 20.43% ± 2.15% apoptosis, respectively; combination with ADM of different concentration (3 µM, 6 µM or 9 µM) and Ophiobolin-O (0.1 µM) induced 42.68% ± 0.55%, 67.22% ± 0.99%, and 71.48% ± 1.20% of cell death, respectively. Therefore, 0.1 µM Ophiobolin-O dramatically strengthened ADM-induced apoptosis in MCF-7/ADR cells (Figure 1D).

### 2.2. ROS Mediated Ophiobolin-O Sensitized G2/M Arrest and Apoptosis of MCF-7/ADR Cells

#### 2.2.1. The Combination Effect of Ophiobolin-O on Apoptotic and Cell Cycle Proteins in MCF-7/ADR Cells

**Figure 2:**
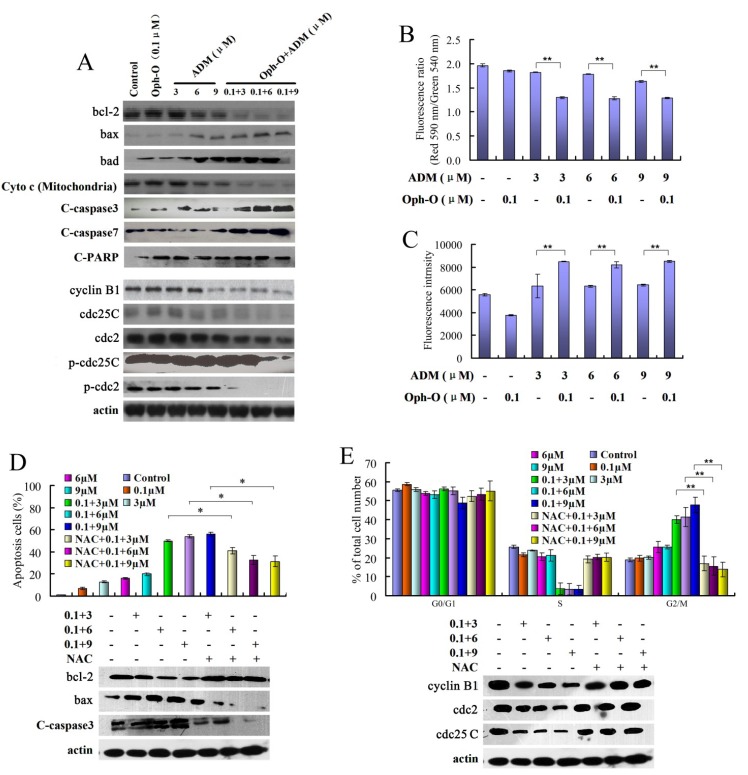
(**A**) Western blot of cells analyzed for bcl-2, bax, bad, cytochrome c (Cyto c) in mitochondria, C-caspase-3, C-caspase-7, C-PARP, cyclin B1, cdc2, p-cdc2, cdc25C and p-cdc25C at 48 h. Results are representative of three separate experiments. β-actin is shown as protein loading control; (**B**) The changes of mitochondrial membrane potential (ΔΨm) and (**C**) ROS level after treated with Ophiobolin-O (0.1 µM), ADM (3 µM, 6 µM or 9 µM) or combination; (**D**,**E**) With or without pre-treatment with ROS inhibitor NAC (10 mmol/L), the changes of apoptosis, cell cycle distribution and related protein levels were showed. Values are means ± SD from three independent experiments. Western blot results from representative experiments were normalized to β-actin expression. * *P* < 0.05, ** *P* < 0.01 *vs.* control group.

Western blot detected the changes of apoptotic proteins, and the results are shown in Figure 2A. After combination treatment with Ophiobolin-O (0.1 µM) and ADM (3 µM, 6 µM or 9 µM), the expression of bax, Cleaved-PARP, bad, Cleaved-caspase3 and Cleaved-caspase7 increased, however, expression of bcl-2 and mitochondrial cytochrome c (Cyto c) decreased, compared to the Ophiobolin-O (0.1 µM) or ADM (3 µM, 6 µM or 9 µM) alone group. Moreover, the combination treatment also caused a significant decrease in the expression of cyclin B1, cdc2, p-cdc2, cdc25C and p-cdc25C (Figure 2A).

#### 2.2.2. The Effects of Combination with Ophiobolin-O on Mitochondrial Membrane Potential of MCF-7/ADR Cells

We used JC-1, which is a widely used probe, to detect the changes of mitochondrial membrane potential (ÄØm) [10,11]. Treatment with Ophiobolin-O (0.1 µM) or ADM (3 µM, 6 µM or 9 µM) did not show a significant decrease in red/green fluorescence ratio. However, combination with Ophiobolin-O (0.1 µM) and ADM (3 µM, 6 µM or 9 µM) caused a significant decrease in red/green fluorescence ratio, which indicated that combination treatment induced the loss of mitochondrial membrane potential in MCF-7/ADR cells, suggesting a mitochondrial apoptosis pathway was activated (Figure 2B).

#### 2.2.3. ROS Was Involved in Reversal Effect of Ophiobolin-O

The ROS level was determined by using DCFH-DA as a fluorescence probe [12,13]. Compared with Ophiobolin-O or ADM treatment group, combination treatment with 0.1 µM Ophiobolin-O and ADM (3 µM, 6 µM or 9 µM) significantly increased ROS level in MCF-7/ADR cells (* *P* < 0.05) (Figure 2C). When pre-treated with ROS inhibitor NAC (10 mmol/L), a decrease of apoptosis (from 54.00% ± 3.21% to 37.32% ± 2.25%) was observed, and the ratio of G2/M phase was also decreased from 41.44% ± 1.50% to 16.32% ± 3.00% (Figure 2D,E). Furthermore, the change of both apoptosis and G2/M phase related protein levels were almost reversed by NAC pre-treatment (Figure 2D,E). These results indicated that ROS was involved in reversal effect of Ophiobolin-O through cell death and cell cycle arrest.

### 2.3. Ophiobolin-O Decreased the Expression of MDR1 and ADM Accumulation via Inhibiting the Activity of the MDR1 Gene Promoter

#### 2.3.1. Ophiobolin-O Down-Regulated MDR1

**Figure 3:**
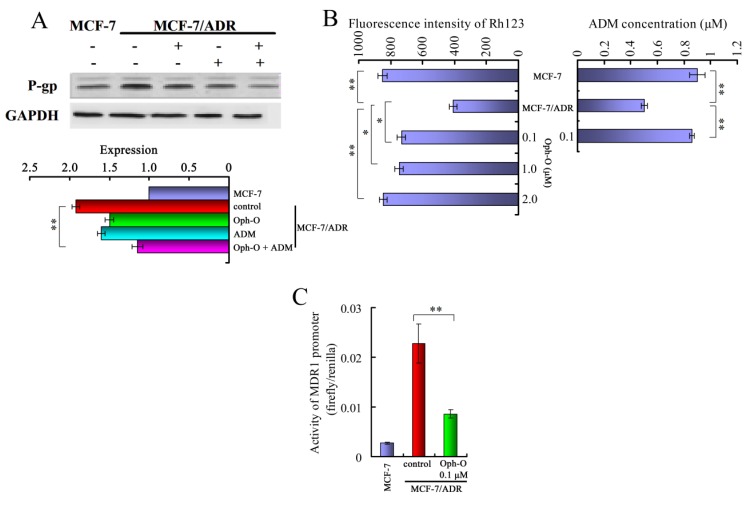
(**A**) Western blotting and real-time PCR showed gene and protein expression of P-gp in MCF-7/ADR cells; (**B**) Control cells and MCF-7/ADR cells treated with 0.1, 1 or 2 µM Ophiobolin-O were collected, the mean fluorescence intensity of retained intracellular Rh123 was examined by measuring with Flow cytometry; Effect of Ophiobolin-O on the intracellular accumulation of ADM in MCF-7/ADR was measured by pre-treatment with 0.1 µM Ophiobolin-O, and compared with MCF-7 and MCF-7/ADR cells, data were expressed as means ± SD of three independent experiments, * *P* < 0.05, ** *P* < 0.01 *vs.* control group (Con group, the untreated MCF-7/ADR cells); (**C**) Cells were co-transfected with recombinant vector pGL3-Basic-MDR1 promoter, dual-reporter gene assays were performed in co-transfected cells with or without treatment of Ophiobolin-O (0.1 µM) and the data are expressed as firefly luciferase/renilla luciferase, data are means ± SD from triplicate experiments.

In order to explore the reversal mechanism of Ophiobolin-O, we treated cells with 0.1 µM Ophiobolin-O and 6.67 µM ADM either alone or in combination for 48 h, the real-time PCR showed that MDR1 gene was significantly inhibited by combination with Ophiobolin-O, and P-glycoprotein were also down-regulated (Figure 3A).

#### 2.3.2. Ophiobolin-O Inhibited Effect of P-Glycoprotein Function in MCF-7/ADR Cells

We used Rhodamine 123 (Rh123), a P-glycoprotein-transported fluorescent dye, to measure the function of P-glycoprotein in MCF-7/ADR cells. The efflux of Rh123 is believed to be proportional to the level of P-glycoprotein expression and can be reduced by P-glycoprotein inhibition [14,15]. We found that the fluorescence intensity of Rh123 in MCF-7/ADR cells was about half of that in the drug-sensitive MCF-7 cells. And 0.1, 1 or 2 µM Ophiobolin-O all significantly increased the fluorescence intensity of Rh123 in MCF-7/ADR cells; no significant difference was observed between these groups, indicating even at low concentration, Ophiobolin-O can also present inhibition effect towards P-glycoprotein function (Figure 3B). We further detected the intracellular accumulation of ADM. As shown in Figure 3C, accumulation of ADM in MCF-7 cells was approximately two times more than that in MCF-7/ADR cells. After the MCF-7/ADR cells were treated with 0.1 µM Ophiobolin-O, the intracellular accumulation of ADM was markedly increased in MCF-7/ADR cells.

#### 2.3.3. Ophiobolin-O Inhibited the Activity of MDR1 Gene Promoter

To explore the mechanism of resistance to ADM in MCF-7/ADR cells, MDR1 promoter, recombinant vector pGL3-basic-MDR1 promoter, was constructed and its activity was determined by vector transient transfection and dual luciferase assay. Following co-transfection of the recombinant vector pGL3-basic-MDR1 promoter and the control vector pRL-SV40 into cells, the MDR1 promoter expression level dramatically decreased when treated with 0.1 µM Ophiobolin-O for 3 h compared to control group (Figure 3C). This result indicated that Ophiobolin-O might decrease the expression of P-gp and ADM accumulation via inhibiting the activity of the MDR1 gene promoter.

### 2.4. Inhibitory Effects on Tumor Growth of Combined Treatment with Ophiobolin-O and ADM in Nude Mice

**Figure 4:**
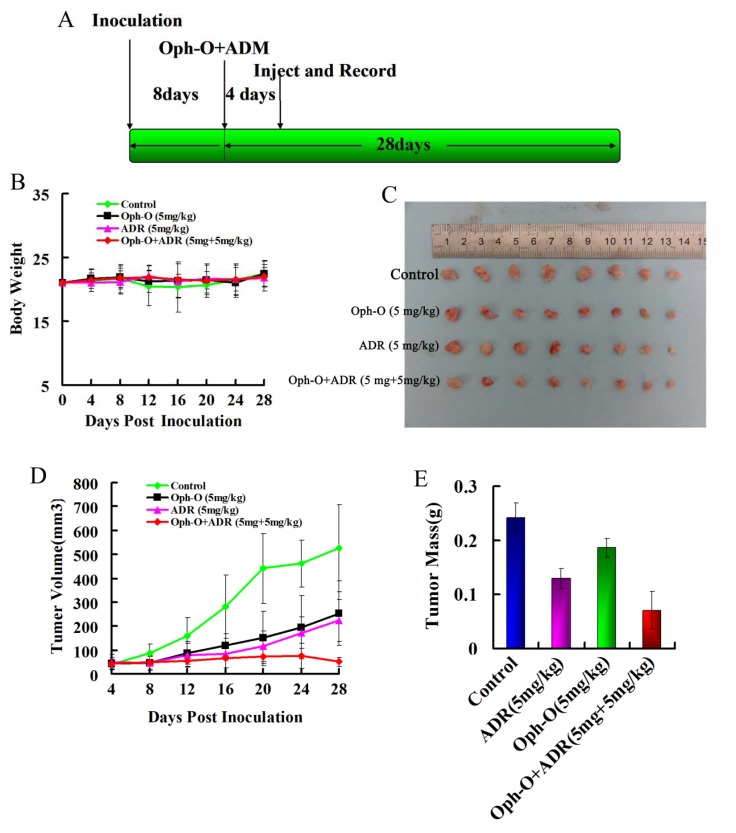
Effect of Ophiobolin-O and ADM on tumor growth in a xenograft model; (**A**) BALB/c female athymic mice were injected with 5 × 10^6^ MCF-7/ADR cells s.c. for the development of subcutaneous tumors, the mice were randomized into four groups (*N* = 8) and treated with Vehicle (1% DMSO), 5 mg/kg ADM or 5 mg/kg Ophiobolin-O i.v. alone, and combination according to the protocol in panel (**A**); (**B**) Changes in nude mice weight; (**C**) Tumor image from various treatment groups; (**D**) Tumor volume measurements; (**E**) Average tumor mass at sacrifice.

To determine whether combination treatment inhibits tumor growth *in vivo*, equal numbers of MCF-7/ADR cells were injected subcutaneously (s.c.) into the right armpit of six-week old BALB/c female athymic mice. Vehicle-treated control mice (1% DMSO) were used as negative control group to assess effect and toxicity. In this study, we adopted the dose of 5 mg/kg intravenously (i.v.), according to the preliminary experiments. Compared with the control group, treatment alone or in combination did not cause a significant change in the weight of nude mice (Figure 4B). However, treatment with Ophiobolin-O (5 mg/kg) or ADM (5 mg/kg) did not significantly inhibit the growth of tumors in nude mice; the inhibition percentage of tumor growth relative to the vehicle control were 46.40% ± 5.20% and 23.17% ± 3.80%, respectively. In contrary, tumor growth inhibition in the combination group was very obvious, and both tumor mass and volume were significantly reduced (Figure 4C–E). Combined treatment with Ophiobolin-O (5 mg/kg) and ADM (5 mg/kg), caused the tumor inhibition rate in nude mice to reach 70.75% ± 5.60%.

### 2.5. Discussion

This study is the first evidence to affirm that Ophiobolin-O reverses ADM resistance *in vitro* and *in vivo* via cell cycle arrest and apoptosis sensitization in MCF-7/ADR cells. Combination with Ophiobolin-O and ADM effectively inhibits MCF-7/ADR cell growth *in vitro* by induction of G2/M phase arrest and cell apoptosis, and inhibits tumor cell growth in a mouse xenograft model.

Ophiobolin-O, which belongs to the family of Ophiobolin, was firstly found from *Aspergillus ustus* 094102 fermentation. Ophiobolin compounds have broad-spectrum antimicrobial activity, such as resistance to nematodes, fungi and bacteria. Ophiobolin-O inhibits the growth of various tumor cells, including liver cancer, lung cancer, prostate cancer and breast cancer cells. We previously reported that Ophiobolin-O induced both apoptosis and cell cycle arrest in MCF-7 cells. It is reported here that low concentration of Ophiobolin-O enhanced the effects of ADM on the drug resistance of breast cancer cells, and the possible mechanisms of this effect were examined.

When MCF-7/ADR cells were treated with 0.1 µM Ophiobolin-O alone, there was no significant cell apoptosis or cell cycle arrest. However, when Ophiobolin-O was combined with different concentrations of ADM, the IC_50_ of ADM in MCF-7/ADR cells decreased from 74.00 ± 0.18 µM to 6.67 ± 0.98 µM. Ophiobolin-O significantly reversed the resistance of MCF-7/ADR cells to ADM. Tumor transplantation experiments in nude mice also showed that treatment with Ophiobolin-O or ADM alone produced tumor inhibition rates of 23.17% ± 3.80% and 46.40% ± 5.20%, respectively. But combined treatment with Ophiobolin-O and ADM gave a tumor inhibition rate of 70.75% ± 5.60%. These results showed a strong reversal effect. Combined treatment with Ophiobolin-O, reversed resistance to ADM in human breast cancer cells *in vitro* and *in vivo*.

ADM is a widely used clinical chemotherapy drug that induces tumor cell apoptosis, but resistance often occurs in a variety of tumor cells with long-term use [13,16,17]. In this study, ADM did not significantly induce apoptosis in MCF-7/ADR cells, but combined treatment of a low concentration of Ophiobolin-O enhanced apoptosis and induced G2/M phase cell cycle arrest. Combination treatment also significantly decreased membrane potential and mitochondrial cytochrome C release in MCF-7/ADR cells. The expression of bax, bad and other pro-apoptotic proteins was elevated, and the expression of the anti-apoptotic protein bcl-2, decreased. Together, these results showed that a low concentration of Ophiobolin-O promoted the mitochondrial apoptotic pathway induced by ADM in MCF-7/ADR cells. Combined treatment with Ophiobolin-O also caused changes in cell cycle-related proteins in MCF-7/ADR cells. The expression of several cyclin proteins, including cyclin B1, cdc2 and cdc25C was down regulated, and cells in G2 phase significantly increased after combination treatment. Combined treatment with Ophiobolin-O also significantly increased ROS in MCF-7/ADR cells. Pre-treatment with the ROS inhibitor NAC decreased the effects of the combined therapy on apoptosis and cell cycle arrest and the corresponding apoptotic and cell cycle proteins, indicating that ROS played an important role in the mechanism of effects due to combination treatment.

The generation of drug resistance in breast cancer cells correlates with increased expression of resistance proteins including MRP, BCRP, and P-gp [18,19,20]. These proteins decrease the accumulation of ADM in the cells; thereby reducing the effects of ADM. Ophiobolin-O reduced the gene and protein expressions of MRP, BCRP, and P-gp, especially P-gp in MCF-7/ADR cells. Reduction of MRP and BCRP were not significant as P-gp (data does not show). Otherwise, ADM and Rh123 were employed to study P-gp transport function, since they are good P-gp substrates with an auto-fluorescence capacity [21]. Figure 3B indicated that Ophiobolin-O, at low concentration, could enhance the accumulation of intracellular ADM and Rh123. These results demonstrated that the Ophiobolin-O could inhibit the transport activity of P-gp, and enhance the sensitivity of MCF-7/ADR cells to cytotoxic drugs. Therefore, we concluded MDR1 gene promoter, which mediated P-gp expression, may be involved in Ophiobolin-O reversal effect. Down-regulation of P-gp expression would increase the concentrations of ADM in cells, thereby promoting ADM-induced apoptosis.

All of the above strongly suggests the combination effects of a low concentration of Ophiobolin-O and ADM in MCF-7/ADR cells decreased the expression of P-gp, increased the concentrations of ADM, promoted apoptosis and cell cycle arrest induced by ADM and inhibited breast cancer cell growth *in vitro* and *in vivo*. ROS was involved in Ophiobolin-O reversal effect through mediation of cell death and cell cycle arrest. Ophiobolin-O has an important clinical significance for the multi-drug resistance of tumor therapy; it can be developed into a new reversal agent for cancer chemotherapy.

## 3. Experimental Section

### 3.1. Compound and Cell Culture

Ophiobolin-O, which belongs to the family of Ophiobolin, is a natural compound that has been isolated from *Aspergillus ustus* 094102. The compound was dissolved in DMSO with a final DMSO solution less than 0.1%.

The human breast cancer cell line MCF-7 was purchased from the cell library of biochemistry and cell biology, Chinese Academy of Sciences Institute, and was cultured in DMEM without phenol red (containing 10% inactivated fetal bovine serum, 100 U/mL penicillin and 100 µg/mL streptomycin). A human breast cancer cell line resistant to ADM, and MCF-7/ADR was purchased from Nanjing Keygen Biotech, and was cultured in RPMI1640 medium (containing 10% inactivated new-born bovine serum, 100 U/mL penicillin, 100 µg/mL streptomycin and 500–1000 ng/mL ADM). Both cell lines were cultured at 37 °C in a 5% CO_2_ incubator. Cells in logarithmic growth phase were used in experiments.

### 3.2. Cell Viability Assay

The cell viability was assayed by MTT [17]. Briefly, cells were harvested during logarithmic growth phase, and seeded in 96-well plates at a density of 1 × 10^6^ cells/mL, compounds were added to wells with the varieties of concentrations. Cell survival rate (%) = [(treated group with drug A550 − blank group A550)/(negative control group A550 − blank group A550)] × 100%. The calculation of IC_50_ value as follow: Growth inhibition rate of each drug concentration group (%) = (1 − experimental OD value/control OD value) × 100%. IC_50_ = lg^−1^[*X*m − *i* (∑P − 0.5)], *X*m: The maximum concentration of numerical design; *i*: The logarithm value of each concentration multiple proportions concentration; ∑P: The sum of every group growth inhibition rate; 0.5: Empirical constants. Values are means ± SD from three independent experiments.

### 3.3. Apoptosis and Cell Cycle Assay

Apoptosis was assessed by labeling cells with annexin V-FITC and PI [22,23], cells were then harvested after treatment. Next, they were washed twice with cold PBS, re-suspended in binding buffer and stained with annexin V-FITC and PI solution (BD Pharmingen). Binding buffer was added, and cells were analyzed by flow cytometry (FACSCalibur, BD Bioscience).

Harvested cells were fixed in 70% ethanol, washed with PBS, incubated with RNase and then stained with PI. The cell cycle phase analysis was performed by using a FACScalibur flow cytometer. Values are means ± SD from three independent experiments.

### 3.4. Real-Time PCR

Total RNA was isolated with the TRIzol reagent of TaKaRa RNAisoTM Plus, and RT-PCR was performed [24]. The primers were as follows: MDR1 sense 5′-AGA AGG TTC TGG GAA GTC GC-3′, anti-sense 5′-AGC ACT GTG TTG GCG TAC AG-3′; GAPDH sense 5′-AAT GCA TCC TGC ACC ACC AA-3′, anti-sense 5′-GTA GCC ATA TTC ATT GTC ATA-3′. Primer synthesis was performed by Shanghai SANGON Biotech Corp. The data analysis was carried out with the Light Cycler Software version 3.5. Values are means ± SD from three independent experiments.

### 3.5. Western Blotting Analysis

Cells were lysed in RIPA buffer (Sigma Chemical Co., St. Louis, MO, USA) and centrifuged for 15 min at 4 °C; the supernatant was transferred to a fresh tube. For western blot analysis, equal amounts of total protein were mixed with SDS sample buffer, incubated at 100 °C for 5 min, and separated by SDS-PAGE. After electrophoresis, protein was blotted onto a NC membrane (Millipore Co., Bedford, MA, USA) and blocked for 1h at room temperature. Each membrane was incubated with the appropriate primary antibodies at 4 °C overnight. The blots were also incubated with the appropriate primary antibodies at 4 °C overnight. The blots were incubated with HRP-conjugated secondary antibodies for 1 h, washed three times with PBST, and visualized by Immobilon Western Chemiluminescent HRP substrate (Millipore Co., Bedford, MA, USA) [25]. The results are representative of three separate experiments.

### 3.6. Rhodamine 123 (Rh123) Efflux Studies

Samples containing 1 × 10^5^ cells/mL were incubated with Rh123 at a final concentration of 0.5 mg/mL at 37 °C for 30 min. After washing, Rh123-stained cells were incubated in a dye-free RPMI medium for additional 90 min for efflux. Flow cytometry was performed for Rh123-associated fluorescence.

### 3.7. Intracellular Adriamycin Accumulation

The cells were treated with 0.1 µM Ophiobolin-O and exposed to 6.67 µM ADM. The control groups were treated with 6.67 µM ADM alone. After incubation for 3 h, cells were washed three times with ice-cold phosphate buffered saline (PBS, pH 7.2). The cells were re-suspended in HCl 0.3 M in 60% ethanol overnight [26], and the mixture was centrifuged for 10 min at 12,000 rpm. The intracellular ADM concentrations were determined by HPLC.

### 3.8. MDR1 Promoter Activity by Vector Transient Transfection and Dual Luciferase Assay

Cells were seeded in of 96-well plates for 24 h until cells reached 90%–95% confluence at the time of transfection. The MDR1 promoter recombinant vector pGL3-basic-MDR1 promoter (0.5 µg/well) was mixed with a control vector (10 ng/well) pRL-SV40. Cells were co-transfected according to the manufacturer’s instructions. After transfection with plasmids, the medium was replaced with 100 µL fresh serum-free RPMI-1640 for incubation overnight. Then cells were washed and lysed. After incubation for 15 min at room temperature, the lysate was centrifuged and the supernatant was harvested and analyzed using a commercial dual-luciferase assay kit (Dual Luciferase Assay System; Promega, Madison, WI, USA). The assay was measured on the GloMax20/20 Luminometer (Promega, Madison, WI, USA) [27,28]. The luciferase value/renilla was analyzed. Values are means ± SD from three independent experiments.

### 3.9. Mouse Xenograft Model

The mouse xenograft model was established by s.c. injection of 1 × 10^7^ MCF-7/ADR cells into the right armpit of six-week old BALB/c male athymic mice (National Rodent Laboratory Animal Resource, Shanghai, China) [29]. The mice were randomized into vehicle control and treatment groups. Vehicle or drugs were administered i.v. every three days until sacrifice. Body weight and tumor size were measured and recorded every three days. The tumor size was measured using an electronic caliper, and tumor volumes were calculated using the formula: length × width^2^/2. The tumor inhibition effect was calculated using the following equation: Tumor suppression (%) = (1 − T/C) × 100. Values are means ± SD from three independent experiments.

## 4. Conclusions

Ophiobolin-O could reverse the resistance of MCF-7/ADR cells to ADM. The combination of Ophiobolin-O and ADM decreased the expression of resistance genes and proteins, increased the intracellular accumulation of ADM in MCF-7/ADR cells, promoted apoptosis and cell cycle arrest induced by ADM, and inhibited breast cancer cell growth *in vitro* and *in vivo*.
